# Evaluation of the anterior segment parameters after Nd: YAG laser Capsulotomy: Effect the design of intraocular lens Haptic

**DOI:** 10.12669/pjms.342.12705

**Published:** 2018

**Authors:** Berkay Akmaz, Fahrettin Akay

**Affiliations:** 1 Berkay Akmaz, MD. Izmir Katip Celebi University, Atatürk Training and Research Hospital, Department of Ophthalmology, Izmir, Turkey; 2 Fahrettin Akay, MD. Izmir Katip Celebi University, Atatürk Training and Research Hospital, Department of Ophthalmology, Izmir, Turkey

**Keywords:** Anterior segment, Myopic shift, 1-piece IOL, 3-piece IOL, Nd:YAG laser capsulotomy

## Abstract

**Objective:**

To evaluate the changes in anterior segment parameters after neodymium–yttrium-aluminum-garnet (Nd:YAG) laser capsulotomy in 1-piece and 3-piece IOLs.

**Methods:**

In an institution, 65 eyes of 65 consecutive pseudophakic patients with posterior capsule opacification underwent Nd:YAG laser capsulotomy. The patients were divided into two groups according to the IOL type. Group-1 consisted of 35 subjects with 1-piece IOL and Group-2 consisted of 30 subjects with 3-piece IOL. Anterior segment parameters were measured with the Sirius rotating camera before, one week and one month after Nd:YAG laser capsulotomy.

**Results:**

Mean age was 72.3±5.2 years in 1-piece IOL and 72.3±6.8 years in 3-piece IOL. There were no statistically significant differences before capsulotomy for IOP, axial length, spherical equivalent, anterior chamber depth, central corneal thickness, anterior chamber angle and anterior chamber volume between two IOL groups. BCVA improved after capsulotomy in both groups (p=0.001). Both IOL groups had statistically significant myopic shift compared with the baseline values (P= 0.03 and P=0.01 resp.). Both IOL groups had statistically significant decrease in ACD, from baseline to the 1st week and 1^st^ month (p=0.04 and 0.03 resp.).

**Conclusion:**

To achieve the highest percentage of refractive and anterior segment stability surgeons may prefer to implant the 1-piece IOL design.

## INTRODUCTION

After a period of successful cataract surgery posterior capsule opacification(PCO) is one of the most vision disturbing problem.^1^ Neodymium–yttrium-aluminum garnet (Nd:YAG) laser capsulotomy is the best choice for treatment of PCO in pseudophakic eyes for many years.^2^ Although it seems to be safe but also it has sight-threatening complications like intraocular pressure (IOP) increase, corneal injury, intraocular lens (IOL) pittings, IOL dislocation, anterior vitreous prolapse, vitritis, pupil blockage, cystoid macular oedema, retinal hemorrhage and retinal detachment.^3^,^4^ The evaluation of the changes in anterior segment parameters after Nd:YAG laser capsulotomy may enable important information about mechanisms of complications and refraction changes. Several studies reported different results in central corneal thickness (CCT), anterior chamber depth (ACD), anterior chamber volume (ACV), anterior chamber angle (ACA) and refraction changes after Nd:YAG laser capsulotomy.^5^-^7^ The Sirius rotating Scheimpflug camera (Sirius, CSO, Florence, Italy) provides rapid quantitative information of the anterior segment.^8^-^10^ It examines the posterior surfaces of the cornea, CCT, ACD, ACV, and ACA. This technique has also good results according to the observer variability.

The characteristics of the IOLs implanted in the capsular bag effect anterior segment parameters by the time.^11^ Design of the IOL (i.e., whether it is 1-piece or 3-piece model) may be the most important characteristic. Haptic of the 1-piece IOLs have the same material with optics and they are in soft manner. Otherwise 3-piece IOLs have rigid haptic material made from poly methyl methacrylate (PMMA). Both types of IOLs are widely used in phacoemulsification surgery.^12^ Postoperative stability and performance of the 1-piece IOLs in the bag are better than 3-piece IOLs.^13^ However, it is not entirely clear whether the two types of IOLs change the anterior segment structure in a similar manner after Nd:YAG laser capsulotomy. This prospective study aimed to investigate the effect of Nd:YAG laser capsulotomy on best-corrected Snellen visual acuity (BCVA), refraction, keratometry, intraocular pressure (IOP), CCT, ACD, ACV, and ACA in patients with PCO. We also wanted to compare the IOL haptic design on anterior segment parameters after Nd:YAG laser capsulotomy with 1-piece and 3-piece hydrophobic acrylic material. To the best of our knowledge, different haptic models of IOL in anterior segment parameters after Nd:YAG laser capsulotomy as measured with the Sirius have not been previously reported.

## METHODS

This prospective study designed as a comparative study consisted of the 65 pseudophakic eyes of 65 patients with visually significant PCO and was cnducted between July 2015 and December 2016. Hospital ethics committee approved the procedures. All participants in the study provide written informed consent. All the pseudophakic subjects were divided into two groups according to the acrylic IOL type 1-piece or three piece. A single surgeon (FA) performed all the cataract surgeries using the Infiniti Vision System (Alcon Laboratories). The technique of the phacoemulsification surgery was a superotemporal clear corneal incision, a continuous capsulorhexis of approximately 4.5 to 5.0 mm, and foldable acrylic IOL implantation in the capsular bag. Patients were implanted a 1-piece (AcrySof SA60AT) or 3-piece (AcrySof MA60AC) acrylic IOL (both Alcon, Inc.). No sutures were used during the surgery. Group-1 consisted of 35 subjects with 1-piece IOL and Group-2 consisted of 30 subjects with 3-piece IOL. The meantime from the surgery was 21.2±6.9 months (range: 10–36) in Group-1 and 22.3±6.5 months (Range: 10–34) in Group-2. All patients had PCO and visual acuity loss (at least two Snellen lines decrement compared to the post-surgery 1^st^ month). Corneal pathology, glaucoma, uveitis, posterior segment pathology, history of previous ocular surgery or trauma, history of using topical/systemic medications, or chronic systemic diseases, which effects the anterior segment dynamics, contact lens use and who were unable to understand the study were excluded.

Each subject underwent a complete ophthalmic examination, including BCVA, slit-lamp biomicroscopy, and Goldman applanation tonometry (GAT, Haag-Streit, Bern, Switzerland) for IOP. Axial length (AL) was measured with a biometer (OcuScan, Alcon, Fortworth, Texas, USA). Dilated fundus examination was performed with 90D lens. These examinations were performed before, one week and one month after Nd:YAG laser capsulotomy. BCVA was converted to the logMAR units for statistical analysis. Refraction was measured by using an auto refractometer (Topcon, RM-KR-8800, Tokyo, Japan) after 40 minutes application of cyclopentolate 1%. Sum of the sphere plus half the cylindrical power was calculated as the spherical equivalent. All refractive and IOP measurements were repeated three times by same examiner (BA). All Nd:YAG laser capsulotomies were performed using a Nd:YAG laser (Ellex Ultra Q Nd:YAG laser, Ellex Medical Lasers Limited, Adelaide, Australia) by the same physician(BA). An Abraham capsulotomy lens was utilized to focus power density on the posterior capsule and an approximately 3.0 to 5.0 mm diameter circular area of the central posterior capsule was cleared by emitting laser energy on the capsule. Energy levels starting from 0.8 miili joules(mJ) and up to 3.6 mJ were used for procedure. Total laser energy and pulse numbers used for capsulotomy were recorded. All patients were advised 1% prednisolone acetate eye drops four times and apraclonidine hydrochloride 0.5% two times daily for a week. There were no complications due to the capsulotomy procedure.

### Sirius Anterior Segment Measurements

The Sirius topography device analyzes the anterior segment structure. It uses a combination of rotating Scheimpflug camera and a Placido-disc. The scanning process acquires a series of 25 Scheimpflug images and one Placido-disc image for analyzing the anterior segment. Anterior surface data from Placido-disk and Scheimpflug camera are combined for examination. Data for the posterior cornea, anterior chamber, anterior lens and iris are obtained from Scheimpflug images of Sirius.^14^ The software version 1.0.5.72 was used on all eyes in the current study. Measurements were performed while the device was brought into focus and the subject’s eye was aligned to the central fixation light. Three measurements were taken carefully for three times by a single examiner (BA) and average results were used for statistical evaluations.

### Statistical Analysis

SPSS for Windows Version 21.0 (IBM Co, USA) was used for statistical analysis. Averages and standard deviations were reported. Shapiro–Wilk test was used to test for normality. The independent *t*-test or Mann-Whitney U test was used according to the normality scores for the groups. Repeated measures analysis of variance (ANOVA) was used to compare the changes in intragroup differences for repeated measurements. Pairwise comparisons were evaluated with the Bonferroni test. *P*<0.05 was accepted statistically significant. Before starting the study, we calculated the required minimum sample size. For this purpose, we used the anticipated mean ACD result in a healthy control group that was found by Gundogan et al.^15^ Mean±standard deviation value was 3.01±0.38 mm. An alpha error of 0.05, and a beta-error of 0.80 was used. A mean value of 3.30 mm as ACD was used to be detected by the statistical analyses. The required sample size was 27. For this reason, we aimed to include at least 27 patients in each group.

## RESULTS

Mean age was 72.3±5.2 years (range: 61–80 years) in 1-piece IOL group and 72.3±6.8 years (range: 59–83 years) in 3-piece IOL group. Mean age was not significantly different between the groups (*P*=0.98). There were no statistically significant differences before capsulotomy for IOP, axial length, spherical equivalent and other anterior segment parameters between two groups (Table-I). As expected, BCVA improved after capsulotomy in both groups. There was statistically significant difference between BCVA after capsulotomy according to the baseline in the two groups (P=0.001). In 3-piece IOL group mean IOP increased at 1^st^ week according to the baseline levels but no statistically significant changes in both groups after capsulotomy.

**Table-I:** Patient characteristics between IOL groups before Nd:YAG laser capsulotomy.

Parameters	1-piece IOL n=35	3-piece IOL n=30	P value
Age (year)	72.3 ± 5.2	72.3 ± 6.8	*0.98
Axial length(mm)	23.42 ± 0.62	22.88 ± 0.93	**0.06
IOP (mmHg)	15.51 ± 2.3	15.93 ± 1.87	*0.67
BCVA (LogMAR)	0.29 ± 0.12	0.32 ± 0.11	**0.27
Spherical equivalents (D)	-0.50 ± 1.01	-0.55 ± 0.92	*0.06
CCT (μm)	530.49 ± 27.29	517.37 ± 22.21	left*0.07
ACD (mm)	3.70 ± 0.32	3.68 ± 0.40	*0.14
ACA (degree)	53.11 ± 6.27	51.93 ± 6.67	*0.46
ACV (mL)	165.89 ± 16.46	164.67 ± 18.38	*0.12

BCVA: Best corrected visual acuity, IOP: intraocular pressure,

logMAR: logarithm of the minimum angle of resolution CCT: central corneal thickness, ACD: anterior chamber depth,

ACV: anterior chamber volume, ACA: anterior chamber angle.

** Mann-Whitney U test,

* Independent t-test

The mean refractive parameter measurements at baseline, 1^st^ week and one month after capsulotomy are sown in Table-II. Both IOL groups had statistically significant myopic shift in spherical error compared with before capsulotomy baseline values (P= 0.03 and P=0.01 resp.). The cylindrical error changes were not significant in both groups. There was minimal myopic spherical equivalent changes in 1-piece IOL group but in 3-piece IOL group had significant myopic shift (P=0.001).

**Table-II:** Comparison of repeated measurements of BCVA, refraction and IOP in 1-piece IOL and 3-piece IOL group.

Parameters	1-piece IOL n=35	*P value	3-piece IOL n=30	*P value
**BCVA (LogMAR)**				
Pretreatment	0.29 ± 0.12	0.001	0.32 ± 0.11	0.001
1 week	0.07 ± 0.05		0.11 ± 0.05	
1 month	0.04 ± 0.05		0.08 ± 0.04	
**IOP (mmHg)**				
Pretreatment	15.51 ± 2.30	0.06	15.93 ± 1.87	0.07
1 week	14.40 ± 2.10		16.03 ± 2.30	
1 month	14.91 ± 1.95		15.10 ± 1.77	
**Spherical error (DS)**				
Pretreatment	0.03 ± 0.95	0.03	-0.07 ± 0.77	0.01
1 week	-0.5 ± 0.72		-0.03 ± 0.75	
1 month	-0.25 ± 0.57		-0.26 ± 0.70	
**Cylindrical error (DC)**				
Pretreatment	-1.06 ± 0.82	0.09	-0.89 ± 0.82	0.41
1 week	-0.98 ± 0.72		-0.73 ± 0.98	
1 month	-0.92 ± 0.70		-0.9 ± 0.79	
**Spherical equivalents (D)**				
Pretreatment	-0.50 ± 1.01	0.17	-0.55 ± 0.92	0.001
1 week	-0.45 ± 0.84		-0.39 ± 0.98	
1 month	-0.60 ± 0.80		-0.72 ± 0.91	

BCVA: Best corrected visual acuity, IOP: Intraocular pressure,

logMAR: logarithm of the minimum angle of resolution.

* Repeated measures ANOVA test.

The mean anterior segment parameter measurements at baseline, 1^st^ week and 1^st^ month after capsulotomy are sown in Table-III. Both IOL groups had statistically significant decrease in ACD, from baseline to the 1st week and 1^st^ month (P=0.04 and 0.03 resp.). In both groups, the changes in CCT, ACA, ACV and keratometry values were not significant.

**Table-III:** Comparison of repeated measurements of the anterior segment parameters in the 1piece IOL and 3-piece IOL group.

Anterior segment parameters	1-piece IOL n=35	*P value	3-piece IOL n=30	*P value
**CCT (μm)**				
Pretreatment	530.49 ± 27.29	0.18	517,37 ± 22.21	0.23
1 week	531.8 ± 27.46		518,33 ± 21.14	
1 month	530.2 ± 26.85		517.66 ± 20.69	
**ACD (mm)**				
Pretreatment	3.70 ± 0.32	0.04	3.68 ± 0.40	0.03
1 week	3.64 ± 0.32		3.58 ± 0.38	
1 month	3.64 ± 0.29		3.60 ± 0.36	
**ACV (mL)**				
Pretreatment	165.89 ± 16.46	0.31	164.67 ± 18.38	0.12
1 week	166.16 ± 15.95		166.6 ± 16.76	
1 month	166.17 ± 20.20		165.97 ± 16.48	
**ACA (degree)**				
Pretreatment	51.97 ± 5.79	0.17	51.93 ± 6.67	0.09
1 week	52.2 ± 6.07		52.6 ± 6.21	
1 month	53.11 ± 6.27		53.13 ± 6.08	
**Keratometry(D)**				
Pretreatment	43.4 ± 1.42	0.66	43.17 ± 1.32	0.06
1 week	43.37 ± 1.44		43.22 ± 1.32	
1 month	43.42 ± 1.43		43.20 ± 1.35	

CCT: Central corneal thickness, ACD: Anterior chamber depth, ACV: Anterior chamber volume,ACA: Anterior chamber angle.

* Repeated measures ANOVA.

## DISCUSSION

Posterior capsular opacification is still the most frequent long-term complication after the cataract surgery. IOL design, material, optic edge profile, haptic material and design seem as the major factors in minimising PCO.^13^,^16^ The decision to perform a capsulotomy is usually based on the patients’ complaints about decreased visual acuity and contrast sensitivity. Intact posterior capsule supports the IOL and sustain the barrier function between the anterior and posterior compartments of the eye. It prevents cystoid macular oedema, retinal detachment and anterior segment neovascularisation.^1^,^17^ Stability of IOL position was also an important problem after Nd:YAG laser capsulotomy for unexpected visual problems. Haptics are the critical to maintain the IOL position.

In the study we found decreased ACD after Nd:YAG laser capsulotomy in both types of acrylic IOLs. Presumably both IOL types shifted forwardly to the anterior chamber after capsulotomy. Optimal cataract surgery aims precise IOL position for best refractive results. Visual quality may spoil due to IOL movement. We evaluated IOL movement by measuring ACD in the study. Several authors have reported no significant changes in ACD measurements after Nd:YAG capsulotomy.^6^,^12^ Khambhiphant reported no significant changes in ACD and SE with IOL Master.^18^ In contrast to our study Findl et al. reported backward movement of the IOL with dual beam partial coherence interferometry.^19^ But similar to our results Öztaşat et al. reported a significant decrease in mean ACD after Nd:YAG capsulotomy, as measured with pentacam anterior segment system.^5^ With using anterior segment optical coherens omography some authors found the increase in ACD, ACV and ACA.^7^,^20^ Different anterior segment analysing systems may be responsible for variations in results. Eventually we think anterior vitreous pressure after capsulotomy may be responsible for anterior IOL movement. Our results showed that 1-piece IOLs moved less than 3-piece IOLs along the optical axis. This may lead to undesired refractive errors.

Refraction changes in our results especially in spherical equivalents (p=0,001) showed that 1-piece IOL group was more stable than 3-piece group. Both groups had myopic shift but spherical equivalents changes in 1-piece IOLs were not significant. Possible mechanism of myopic shift and forward movement of the IOLs may be related to positive vitreous pressure, disruption of capsule integrity and haptic design. According to the results this might be the first study to show that 1-piece IOLs may yield better refractive outcomes than 3-piece IOLs after Nd:YAG capsulotomies. There was a minimal increase in ACV and ACA in both groups but not significant. These results showed that 3-piece IOL group was more unstable as compared to the 1-piece IOL group. Zhong reported 1-piece IOLs exhibits better axial stability and more stable refractive outcomes after three months in the bag.^11^

Some studies have showed that haptic angulation of the 3-piece IOL gradually decreased after a period from cataract surgery. It caused the IOL to shift forward as well as a myopic shift. It was thought that loss of haptic memory changed the haptic angulation and responsible for postoperative forward movement of the IOL.^21^,^22^ Behrouz et al. reported forward shift in both IOL types after phacoemulsification postoperative at 3^rd^ month. They also found, the 1-piece acrylic foldable IOL showed little axial movement and provided stable refraction and the 3-piece IOL had significant forward movement and led to a myopic shift within three months postoperatively.^23^ Eventually these studies reveals the forward shift of the both IOLs after custom cataract surgery without performing Nd:YAG laser capsulotomy.

### Limitations of the study

Firstly, a 1-month follow-up time may be insufficient to detect long-term anterior segment differences between the two IOL models after capsulotomy. Secondly, the sample size was relatively small and larger studies are needed to confirm our results. Thirdly we did not evaluate the tilt and decentration of the IOLs. It may affect the refractive errors. Despite these limitations our results are helpful for further investigation about different haptic designs.

This may be the first report of different refractive and anterior segment outcomes between 1-piece IOLs and 3-piece IOLs, with the latter showing higher accuracy. To achieve the highest percentage of refractive and anterior segment stability surgeons may prefer to implant the 1-piece design. Loss of haptic memory may affect the haptic angulation and we think it is responsible for more forward movement after (Nd:YAG) laser capsulotomy. Further studies are needed for innovations of the better haptic material and design in 3-piece IOLs.

### Author`s Contribution

**BA and FA** conceived, designed and did statistical analysis & editing of manuscript.

**BA and FA** did data collection and manuscript writing.

**FA** did review and final approval of manuscript.
